# Fast, lightweight BBB permeability predictions for molecular design

**DOI:** 10.1039/d6me00041j

**Published:** 2026-11-03

**Authors:** Hengjian Jia, Gabriele C. Sosso

**Affiliations:** a Department of Chemistry, University of Warwick Coventry CV4 7AL West Midlands UK g.sosso@warwick.ac.uk

## Abstract

The blood–brain barrier (BBB) is a selectively permeable border that regulates the transport of chemical compounds into and out of the central nervous system (CNS). To discover medicinal drugs that can target the CNS, we must be able to efficiently screen pools of potential candidate CNS-targeting drugs to identify those that can permeate the BBB. However, drugs that readily permeate the BBB are comparatively rare, thus CNS drug discovery typically requires screening very large candidate libraries. In the last two decades, a diverse portfolio of machine learning models has been developed to predict the permeability of the BBB. However, many of the most accurate models reported to date do not scale well to large screenings, because they depend on high-cost descriptor pipelines and/or computationally intensive model architectures. In this work, we used a straightforward, computationally efficient set of descriptors in conjunction with a lightweight random forest model to perform a large-scale screening for BBB permeable drugs. Specifically, we predicted the propensity to cross the BBB for roughly 117 million compounds from PubChem. The screen ran on CPU-only hardware (64 cores) and completed in less than 800 CPU hours; the main resource constraint was memory, with ∼4 TB RAM required for data handling. From these predictions, we prioritised a small set of top-ranked compounds and used well-tempered metadynamics with lipid-bilayer proxies to assess whether their membrane-crossing barriers were consistent with the model-predicted permeability classes. We hope that similar workflows can be adopted in the near term to improve the throughput of BBB-permeability prioritisation in drug discovery.

## Abstract 2

We screen ∼117 million PubChem compounds for predicted blood–brain barrier permeability using a lightweight fragment-based random forest, then assess selected candidates with lipid-membrane metadynamics.

## Graphical abstract

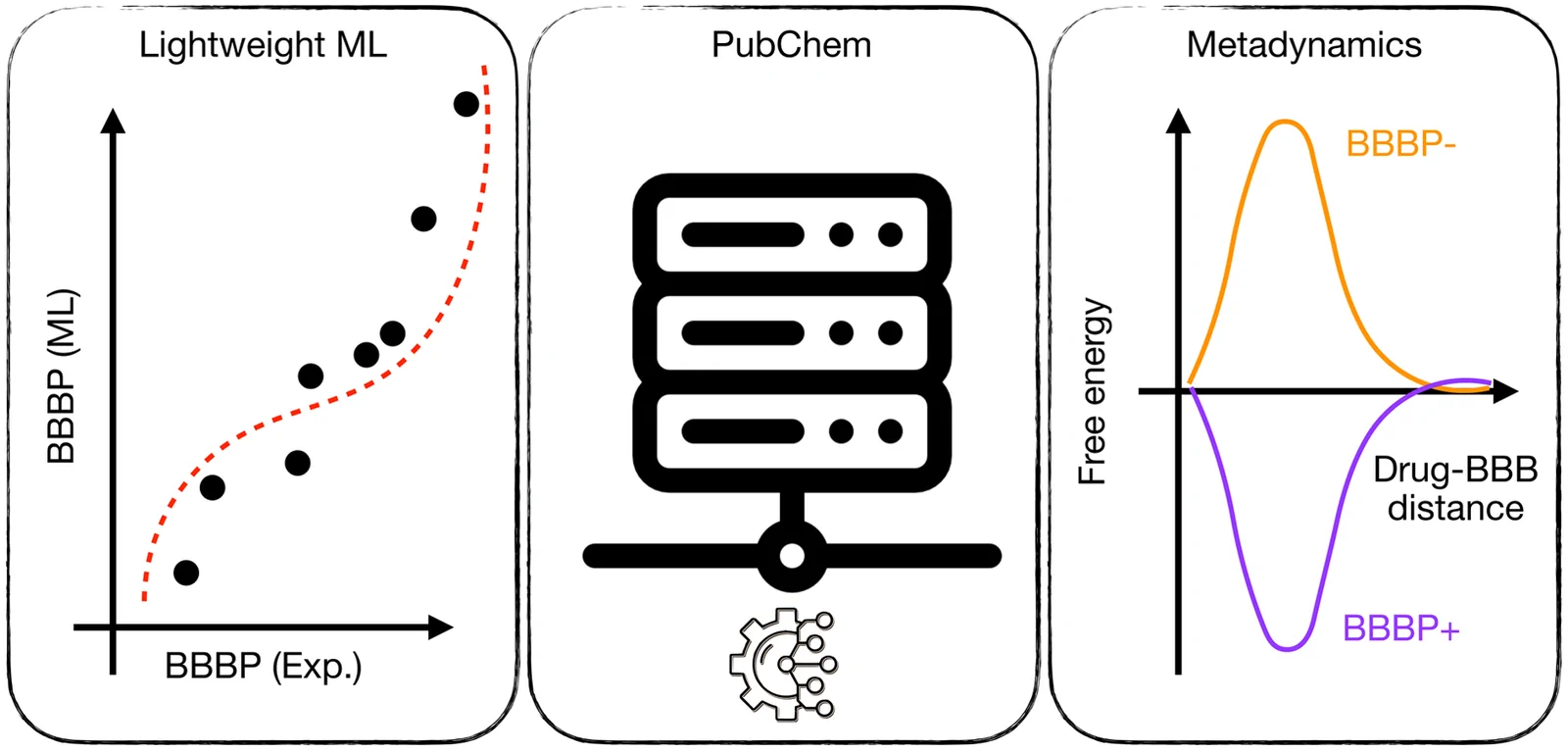

**Design, System, Application**

We combine two complementary design filters to prioritise small molecules for potential blood–brain barrier penetration. An inexpensive fragment-based classifier first screens a very large chemical library, while atomistic simulations probe the thermodynamic cost of crossing lipid membranes for selected candidates. The design objective is to identify molecules that merit closer investigation without making expensive simulations the bottleneck of early-stage screening. The central constraints are the limited chemical coverage of the machine-learning training data, the coarse molecular information retained by the descriptor, and the use of restrained lipid-bilayer proxies rather than the complete biological barrier. Under this protocol, compounds classified as less permeable tend to encounter larger summed membrane-crossing barriers. The workflow is therefore useful for prioritisation of *in vivo* brain exposure, and could guide subsequent experimental tests and more detailed calculations in CNS-directed molecular design.

## 1 Introduction

Neurological disorders are the leading cause of disability worldwide, affecting more than 3 billion people in 2021:^1^ this highlights the urgent need for effective therapeutics. However, the development of drugs capable of reaching the brain remains a major challenge. The transport of chemical compounds between the bloodstream and the central nervous system (CNS) is controlled by the blood–brain barrier (BBB).^2,3^ The BBB also protects the CNS by acting as a selective interface and preventing foreign substances from affecting brain function and homeostasis.^3,4^ As a result, BBB-permeable compounds are relatively rare. An estimated 2–6% of small chemical molecules can permeate the BBB. Larger molecules such as antibodies are effectively completely blocked by the BBB.^5–8^ In fact, recent reports indicate that less than 10% of drugs for neurological diseases enter clinical trials due to poor brain penetration. Thus, to find effective BBB permeable drugs, we may need to screen a large number of potential molecules.^5,7,9^ In turn, the ability to predict BBB permeability for potential new medicinal drugs within the early stages of the pharmaceutical pipeline would be highly valuable.

Whilst *in vitro* high throughput screening methods have been used to decrease the scale of the screening required to find BBB permeable drugs,^9^ these methods remain relatively expensive. A single high throughput screening campaign can cost hundreds of thousands of dollars.^10^ An alternative to *in vitro* screening methods is to use *in silico* methods to predict the BBB permeability of druglike molecules. These approaches include both molecular dynamics (MD) simulations and machine learning (ML) driven methods.^11–13^ Part of the development of MD driven approaches have been fueled by the creation of experimentally validated BBB models such as the framework of Wang *et al.*^14^ However, MD-based methods to assess BBB permeability are typically very computationally intensive.^11,15^ As a result, they tend to be used to study specific molecules, such as AZD3759, or erlotinib and JCN037 as in the case of Liang *et al.*^16,17^ Ianos *et al.* used MD simulations to study the BBB permeation of specific nanoplastics instead (polyethylene, polypropylene, polystyrene and polyethylene terephthalate).^18^ It follows that MD approaches typically cannot be used to screen large number of chemical compounds in an efficient fashion.

ML-driven methods are normally much computationally cheaper than MD-driven approaches. However, ML-driven methods often involve convoluted algorithms leveraging a large set of descriptors (or input features, or fingerprints) generated by, *e.g.*, computational chemistry packages, which may result in computational frameworks that are difficult to scale up for the purposes of large-scale screening. For instance, Faramarzi *et al.*^19^ have constructed a model which correlates 386 features with BBB permeability. These features have been generated using the Leadscope Enterprise and CASE Ultra softwares^20,21^ on a dataset of 921 compounds. In recent years, thanks to developments in transformer based models,^22^ large language models such as those based on the BART model^23^ have begun to be used. Specifically, Huang *et al.*^24^ have adapted the MegaMolBART model from NVidia^25,26^ to predict BBB permeability. Whilst Huang *et al.*^24^ achieved good results, the computational requirement of MegaMolBART is substantial,^26^ and scaling it up to generate predictions on large datasets would be prohibitively costly. Another popular, but complex and computationally expensive approach are ensemble model, such as the model by Feng *et al.*^27^ Their model used a complex ensemble of transformers, random forests, convolutional neural networks and additional algorithms to predict BBB permeability.^22,28,29^ Such ensemble methods would also likely prove challenging to scale to large scale screening attempts. An additional, pivotal challenge in the context of utilising ML-driven methods for BBB permability predictions is that they can struggle to provide any physical insight into the molecular-level details that are responsible for the tendency of a given drug molecule to cross, or not, the BBB. In other words, many ML models are not interpretable, which in turn prevents us from identifying practical design rules to be implemented within the experimental pipeline.^30–32^

In this paper, we utilise a minimalistic ML model (based on a simple and interpretable set of descriptors proposed by Jia and Sosso^31^) to data-mine almost the entirety of the PubChem database (∼110 million chemicals) for compounds which are either likely to be highly BBB permeable or highly BBB nonpermeable. A screening of this scale is (to our understanding) unprecedented in the context of BBB permeability predictions. Due to the minimalistic nature of this model, less than 800 CPU hours were needed to process the entirety of this substantial database. The results of these screening are publicly available *via* Zenodo.^33^

As the original ML model was trained on a relatively small number (∼8000) of datapoints, it is both necessary and interesting to assess its transferability on the much larger and more diverse data set offered by PubChem. To assess the physical consistency of the ML predictions, we used well-tempered metadynamics^34,35^ to investigate 20 previously unseen PubChem compounds: 10 predicted to be strongly BBB-permeant and 10 predicted to be strongly BBB-impermeant. Metadynamics is an enhanced-sampling method that provides a physics-based consistency check on the ML-selected compounds: the simulations use a model independent of the ML descriptors and training data, but the selected compounds do not constitute an external statistical validation set. Under the restrained membrane-crossing protocol used here, the BBBP^−^ candidates tend to exhibit higher total permeation barriers than the BBBP^+^ candidates. This class-level agreement supports the proposed screening strategy without establishing the quantitative accuracy or general transferability of its individual predictions. As such, this combined ML–MD workflow provides a practical route for prioritising compounds for more detailed computational and experimental permeability studies. This provides a practical, scalable prioritisation tool that can accelerate high-throughput screening by focusing future experimental efforts on the most promising BBB-permeable candidates.

The remainder of the paper is organised as follows. Section 2 describes the methodology: section 2.1 outlines the large-scale PubChem screening workflow using the cliques-based random forest model, section 2.2 describes the molecular-dynamics system setup, and section 2.3 details the well-tempered metadynamics protocol used to estimate permeation free-energy barriers. section 3 presents the results and analysis, with section 3.1 reporting the screening outcomes and section 3.2 reporting the metadynamics validation results. Finally, section 4 summarises the main conclusions and discusses limitations and future directions.

## 2 Methods

### 2.1 Screening with random forests

We have decided to apply the ML model developed in ref. 31 (described below) to predict the BBB permeability of the chemical compounds within the PubChem dataset, which as of 2024 contains more than 100 million unique chemical structures. The relevant computational pipeline is illustrated in Fig. 1. As a first step, we preprocessed the PubChem dataset using a similar method to the method used to curate the B3DB dataset.^36^ This is the dataset the ML model was originally trained on. We start by removing: (a.) all compounds that contain “heavy atoms” (*i.e.*, atomic species characterised by an atomic number greater than 20); (b.) all molecules with molecular weight above 1000 g mol^−1^, and (c.) all molecules which cannot be processed by RDKit. This is on order to avoid molecular species that are too dissimilar to our original training data; in addition, very large molecules are almost always blocked by the BBB in any case.^6^ This preprocessing step reduced the roughly 117 million molecules in the PubChem dataset to about 112 million molecules.

**Fig. 1:**
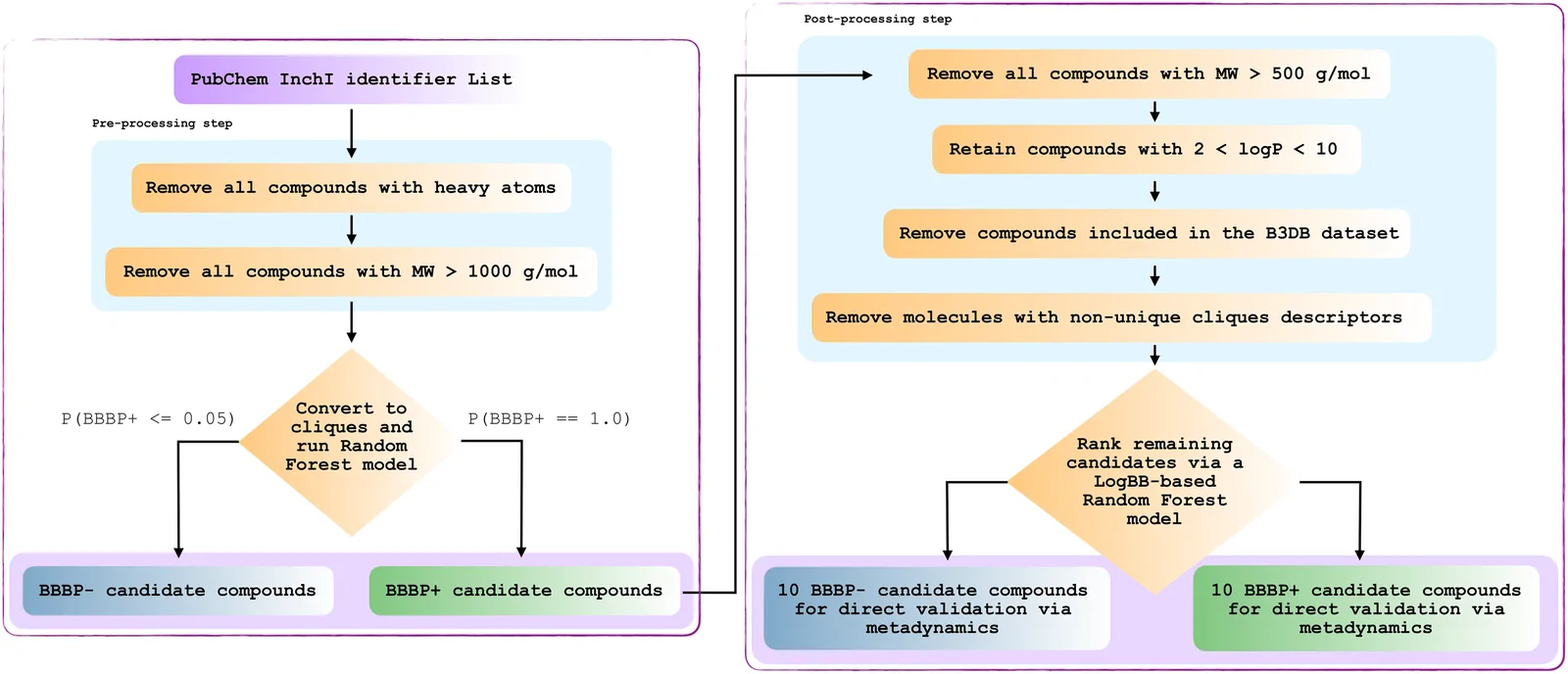
Flowchart illustrating the process of (a.) predicting the BBBP of all the chemical compounds within the PubChem database (InChI identifier list); (b.) selecting a small subset of molecular species for direct validation of their BBBP predictions *via* molecular dynamics (MD) simulations. This includes preprocessing and postprocessing steps.

The resulting data is then converted into an appropriate input and fed to a random forest model^28^ with 128 trees trained on B3DB,^36^ as developed by Jia and Sosso.^31^ More specifically, Jia and Sosso adopted the method of rapidly decomposing molecules into molecular fragments called “cliques”. The cliques are then used to create a vocabulary of all known cliques from the training set, which in this case is the B3DB dataset.^36^ Each clique can then be encoded into a one-hot vector, which allows us to represent molecules as sums of the one-hot vectors corresponding to the cliques present.^30,31^ This process is illustrated in Fig. 2. Note that this descriptor contains information about the presence (or lack thereof) of specific functional groups within a given molecular structure, but it does not provide any information as to where these moieties are positioned within the molecular structure. In other words, the cliques descriptor provide a simplified, transparent description of the “chemistry” of the system alone. This approach turned out to be quite efficient^31^ for the purposes of developing a predictive model for BBB permeability, but it is clearly not suitable for chemical problems (*e.g.*, solubility or physical stability) where structural information plays an important role.

**Fig. 2:**
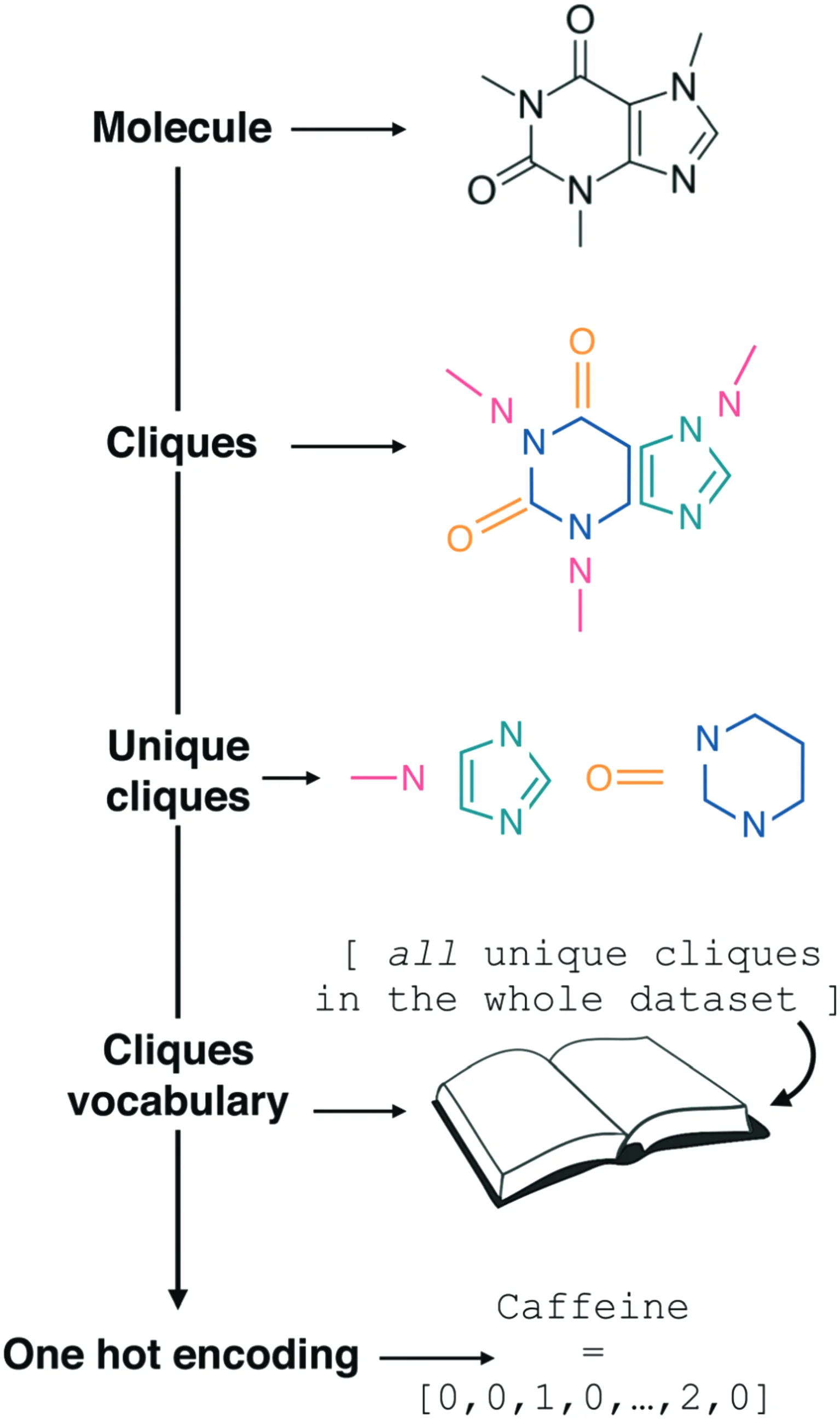
Schematics of the process of generating the “cliques” descriptor. Adapted from Jia and Sosso^31^ and Bernard *et al.*^30^

We classified molecules as BBB permeable (BBBP^+^) or impermeable (BBBP^−^) based on the scores returned by the random forest model. Because the model exhibits a slight bias toward BBBP^+^ predictions (*i.e.*, higher sensitivity than specificity), we adopted conservative selection thresholds: compounds were labelled BBBP^+^ only if their model score was 1.0, and BBBP^−^ if their BBBP^+^ score was ≤0.05. Note that these scores should not be interpreted as physical probabilities of BBB permeation. In particular, the BBBP^+^ score is obtained from RandomForestClassifier.predict_proba(), and a value of 1.0 denotes the maximum support returned by the 128-tree ensemble. We use this value as a (stringent) selection criterion.

The trained random forest model is then applied to the PubChem dataset after converting the compounds to the same clique-based descriptor representation. This model is a binary classifier that outputs a classification score for BBB permeability (not a calibrated physical permeation probability). The results are available *via* Zenodo.^33^ This process produces almost 200 000 BBBP^+^ and 97 000 BBBP^−^ candidates, far more than we can reasonably validate and investigate by means of molecular dynamics simulations. Thus, we further refined this selection by a applying a sequence of filters, illustrated in Fig. 1 and 3. Specifically, we first discarded all molecules with molecular weight above 500 g mol^−1^ and with log *P* > 10 or log *P* < 2 (calculated using RDKit), as these molecules are unlikely to be considered drug-like, based on Lipinski's rule of five.^37,38^ We also cross-referenced our data against the DrugBank^39^ and the B3DB^36^ datasets and removed any molecules which have been previously studied, so as to limit our selection to “novel” molecular sepcies. Finally we removed any molecules not uniquely represented by Jia and Sosso's cliques descriptor.^31^ This narrowed our candidates list down to roughly 4000 BBBP^+^ candidates, and 1000 BBBP^−^ candidates.

**Fig. 3:**
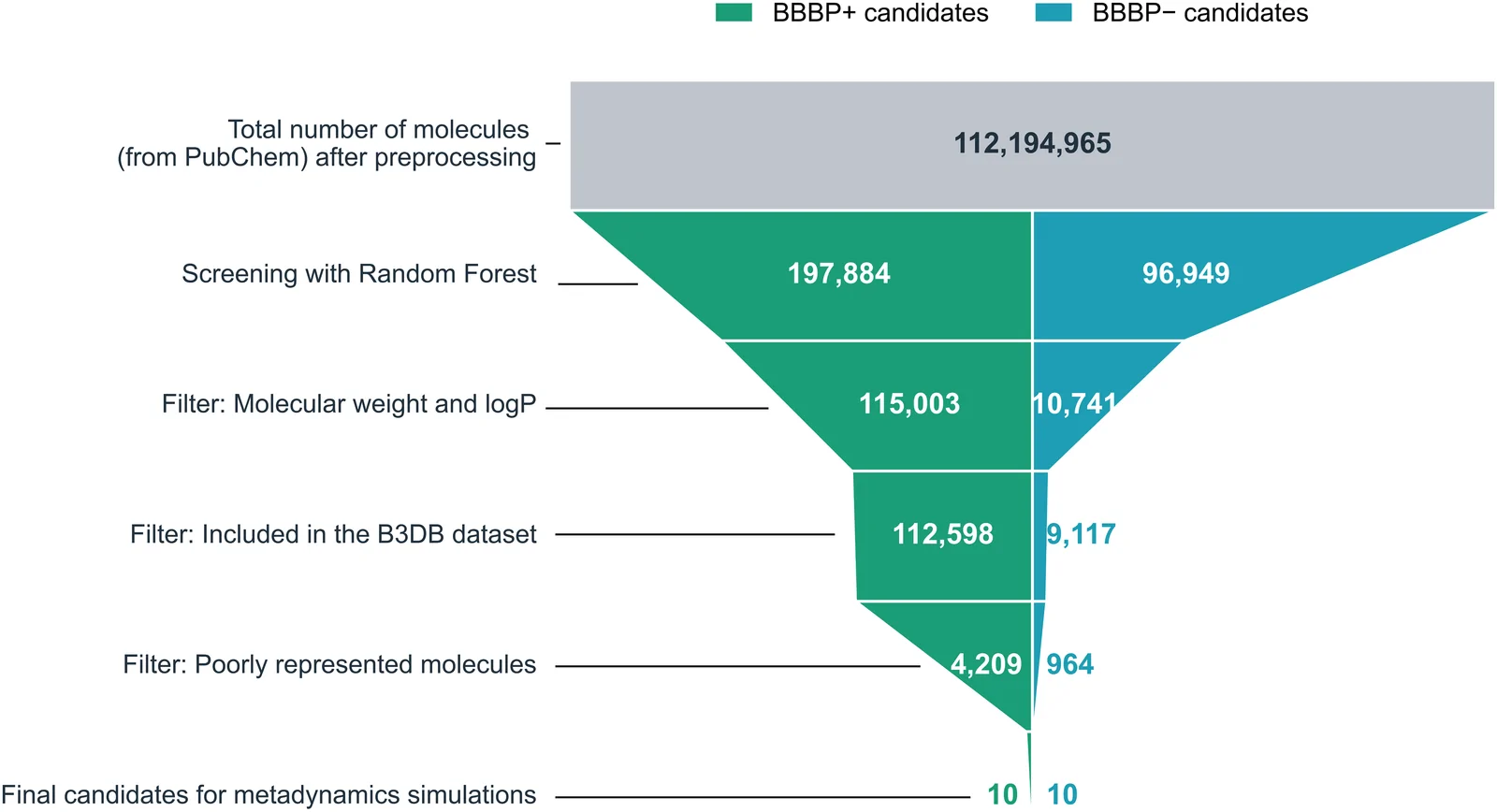
Funnel chart of the filtering pipeline used to reduce the PubChem candidate set to a small pool of compounds selected for simulation-based validation of ML BBB-permeability predictions *via* well-tempered metadynamics (see main text). Counts are plotted on a logarithmic scale.

After filtering, we retained roughly 4000 BBBP^+^ and 1000 BBBP^−^ candidates – still far too many for feasible validation *via* molecular dynamics. Rather than selecting compounds at random for simulation, we introduced a second model previously trained on B3DB as a regression predictor of log BB, which we used to further prioritise candidates. Note that log BB is a continuous metric of BBB permeability defined as 
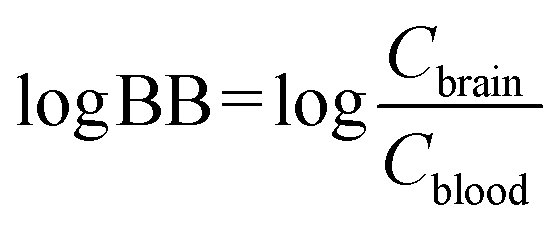
 where *C*_brain_ is the concentration of the molecule found within the brain, and *C*_blood_ is the concentration of the molecule within the blood (outside the brain). The model is used only to order the already filtered BBBP^+^ and BBBP^−^ candidate pools for the purpose of selecting compounds for simulation; as the model explains less than half of the variance in the available log BB data (*R*^2^ = 0.494), its output should not be interpreted as an accurate, quantitative prediction of log BB.

From the filtered pools, we used the log BB ranking to select 10 BBBP^+^ compounds from the high-score end of the distribution and 10 BBBP^−^ compounds from the low-score end for validation by means of well-tempered metadynamics.^34,35^ Here, “validation” refers to a physics-based assessment performed with a methodology independent of the ML model; because the 20 compounds were selected by the ML pipeline, they do not constitute an external validation set in the strict statistical sense. In practice, we also required that each compound could be parametrised with CGenFF^40^*via* CHARMM-GUI^41,42^ with acceptable penalty scores. Molecules associated with high penalties (indicative of unreliable parameters), or that failed during topology generation or initial equilibration, were discarded and replaced by the next-ranked candidates. During this process, several azetidine-containing candidates produced unstable trajectories, with GROMACS failing within the first few integration steps, which we attributed to difficulties in their CHARMM-GUI/CGenFF parameterisation. We therefore excluded compounds containing the azetidine moiety (*i.e.*, a four-membered saturated ring containing one nitrogen atom) from both the BBBP^+^ and BBBP^−^ candidate pools before finalising the validation set. In practice, the problematic compounds encountered during parameterisation happened to belong to the BBBP^−^ shortlist only; none of the BBBP^+^ candidates entering the parameterisation shortlist contained this particular motif.

### 2.2 Molecular dynamics simulations

To perform molecular dynamics simulations, we utilised the GROMACS MD package (version 2023.3)^43^ in conjunction with the CHARMM36 (July 2022 version)^40^ force field. Specifically the CharmmGUI^41,42^ and GROMACS 2023.3 (ref. 43) interface were employed to generate the topology of the systems, which include the drug-like molecule, a lipid bilayer as a proxy of the BBB and water. These three components were modelled *via* the CGenFF force field, the CHARMM36 force field and the TIP3P water model,^44^ respectively. As discussed in the previous section, we have selected 10 BBBP^+^ and 10 BBBP^−^ candidates according to their predicted log BB.

The BBB is, in reality, an exceptionally complex biological system. For the purposes of assessing the tendency of a given molecule to cross the BBB *via* atomistic MD simulations, we are forced to use a lipid bilayer as a proxy of the actual BBB. This is a common approach which, perhaps surprisingly, has yield rather insightful results within the recent literature, see *e.g.* ref. 11, 14, 16, 17 and 45. Here, we utilised the BBB model developed by Wang *et al.*^14^ Note that we do differentiate between apical and basolateral membranes. The apical membrane faces the external environment (*i.e.*, towards the lumen), whilst the basolateral membrane faces the CNS (*i.e.*, away from the lumen), and it is thus in contact with neighbouring cells as well as the extracellular matrix.^14,46^ Importantly, the composition of these two classes of membranes is markedly different, hence why we have chosen to explicitly include both within our validation protocol.^14^

Thus, for each of the 20 molecules previously selected for the MD simulations validation stage, we constructed 2 independent systems, one for each membrane considered: one featuring the apical membrane, and the other featuring the basolateral membrane. The exact compositions of the bilayers are reported in Table 1. In each simulation box, we surrounded each side of the membrane with a layer of water (approximately 6 nm thick). This layer of water was essential to monitor the path of the drug-like molecule from the bulk of the water phase through the membranes. This specific setup allowed us to observe a clear change of free energy of the system (with respect to the collective variable of choice, as we will discuss shortly) as the molecule approached the membrane. We add sodium and chlorine ions to the water at a concentration of 0.15 mol per litre to mimic the physiological conditions of tissue fluid. For both types of membranes, this procedure results in a orthorhombic simulation box roughly 6.8 nm wide in the *xy* plane, and 17.2 nm tall along the *z* axis. The membrane lies in the centre of the simulation box. Periodic boundary conditions were applied in all three Cartesian directions. Although fully periodic setups can introduce finite-size artefacts (most notably spurious interactions between periodic images of the membrane/water slabs along the bilayer normal) we retained this choice to be consistent with the simulation protocol previously used by Wang *et al.*^14^

**Table 1:** Composition of the lipid bilayers utilised as proxies of the BBB in our MD simulations. Note the distinction between apical and basolateral membranes, as discussed by Wang *et al.*^14^ lipid residues are named according to the CHARMM36 force-field conventions

Molecule/ion	Residue name	Apical	Basolateral
Cholesterol	CHL1	56	56
3-Stearoyl-2-arachidonyl-d-*glycero*-1-phosphatidylcholine	SAPC	16	12
1-Stearoyl-2-linoleoyl-phosphatidylcholine	SLPC	16	12
1-Stearoyl-2-arachidonyl-phosphatidylserine	SAPS	16	20
Sphingomyelin d181/181 w/oleic acid	OSM	36	4
1-Stearoyl-2-arachidonyl-phosphatidylethanolamine	SAPE	28	28
1-Stearoyl-2-oleoyl-phosphatidylethanolamine	SOPE	12	12
Phosphatidylinositol	SAPI	4	4
3-Palmitoyl-2-oleoyl-d-*glycero*-1-phosphatidylcholine	POPC	8	4
Sodium ion	SOD	73	77
Chlorine ion	CLA	53	53
Water (TIP3 model)	TIP3	19 968	19 674

Starting from a simulation box that contained only the membrane slab and the water phase (plus the ions), we first performed an energy minimisation using steepest descent for each membrane simulation. We used a convergence limit of 1000 kJ mol^−1^ nm^−1^ for the maximum force acting on any atom within the system. To constrain the bond lengths of all covalent bonds involving hydrogen, we utilised the LINCS algorithm.^47^ The SETTLE algorithm^48^ was used to constrain the geometry of TIP3 water molecules. Each membrane system was equilibrated through a sequence of NVT and NPT simulations at 310 K. The equilibration began with two consecutive NVT runs of 125 ps each, with positional an dihedral restraints on the membrane lipids with force constant 1000 kJ mol^−1^ nm^−2^ and 400 kJ mol^−1^ nm^−2^ respectively. Both NVT runs used a leap-frog integrator with a 1 fs timestep. For temperature coupling, we used the velocity rescaling thermostat (v-rescale in GROMACS) with a coupling constant of 1.0 ps.^49^ Following the recommended CHARMM36 settings for GROMACS, we used a 1.2 nm cutoff for both van der Waals and short-range electrostatic interactions. The system was then equilibrated within the NPT ensemble, using a stochastic cell scaling barostat (C-rescale in GROMACS)^50^ with a coupling constant of 5 ps and 1 bar reference pressure. This equilibration was completed in four stages: 125 ps with a 1 fs timestep and 400 kJ mol^−1^ nm^−2^ restraint, followed by 500 ps with 2 fs timestep and 200 kJ mol^−1^ nm^−2^ restraint, then 500 ps with 2 fs timestep and 40 kJ mol^−1^ nm^−2^ restraint, and finally 100.5 ns without any restraints, to ensure the full equilibration of the system. This procedure yielded two well-equilibrated bilayer systems representing the apical and basolateral membranes, which were subsequently used as starting configurations for all production simulations.

For each candidate, the drug molecule was inserted into the aqueous phase above the membrane at *xyz* coordinates corresponding roughly to (3.4, 3.4, 16.5) nm, removing water molecules that sterically clashed. In cases where our candidate drug molecule was characterised by a non-zero total charge, we also added the counter-ions necessary to neutralise the overall charge of the system. Following this step, each system was further equilibrated through a short NVT run and a longer NPT run, using the same thermostat, barostat and all other configurations as in the previous stage. To be specific, the NVT equilibration was performed for 10 ns with a 1 fs timestep using a leap-frog integrator, followed by a 100 ns NPT equilibration with a 2 fs timestep to ensure full relaxation in the presence of the drug molecule. During this stage, the membrane was restrained in the *Z* axis with a force constant of 200 kJ mol^−1^ nm^−2^ to prevent displacement from the centre of the simulation box due to interactions with the inserted molecule. In addition, we used PLUMED^51^ version 2. 9 (ref. 52) to restrain the candidate drug molecule laterally around the coordinate (*x*, *y*) = (3.4, 3.4) nm *via* a harmonic restraint with spring constant 100 kJ mol^−1^ nm^−2^. Prior to the metadynamics simulations, the molecule was positioned at *z* = 16.5 nm, in the middle of the aqueous slab and sufficiently far from the membrane that solute–membrane interactions were negligible. This provided a simple and reproducible bulk-water reference state from which to initiate the permeation calculation. During the production metadynamics simulations, the solute was free to move along the bilayer normal, while the restraint in the *xy* plane defined the local membrane-crossing path. A representative snapshot illustrating this computational setup is shown in Fig. 4.

**Fig. 4:**
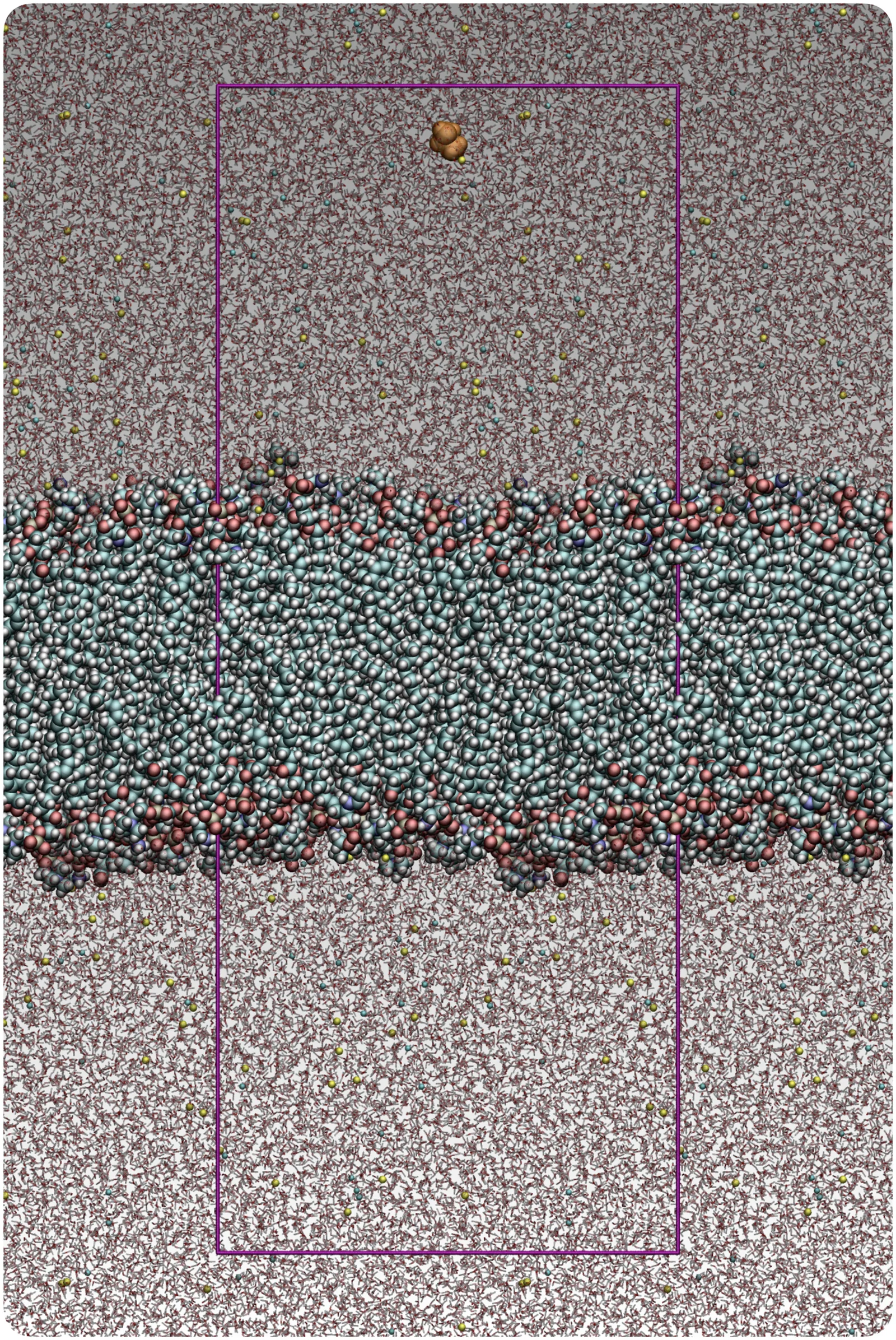
A representative snapshot of our MD setup. The drug molecule (isopropanol, in this example) can be found (highlighted in orange) in the upper section of aqueous phase. The lipid bilayer is centred in the simulation box and surrounded by water and ions, to mimic physiological conditions.

### 2.3 Well tempered metadynamics

After equilibration, we carried out well-tempered metadynamics to quantify membrane permeation using GROMACS 2023.3 coupled to PLUMED 2.9. Production metadynamics was run in the NVT ensemble, rather than NPT, to keep the box dimensions fixed. This is because we found that drug–membrane interactions can drive sizeable pressure fluctuations: under NPT conditions (stochastic cell-rescaling barostat), such fluctuations led to transient box deformations that complicate interpretation of the solute-membrane separation and ultimately the resulting free-energy profiles. We recognise that constraining the dynamics to NVT is a limitation; here, the metadynamics simulations are used primarily to rank and compare candidates under a single, consistent protocol, rather than to deliver definitive absolute permeabilities. This choice makes the protocol robust and tractable for the full validation set (20 compounds evaluated against two BBB membrane proxies), *i.e.* 40 metadynamics simulations that must each be converged to extract reliable trends.

To assess the effect of fixing the membrane area, we also repeated the metadynamics calculations for the five reference compounds using C-rescale pressure coupling in the membrane plane. The *xy* compressibility was 4.5 × 10^−5^ bar^−1^, while the *z* compressibility was set to zero. This allows the membrane area to relax while retaining a fixed box length along the permeation coordinate. We chose this setup to isolate the effect of lateral area relaxation without introducing changes in the box height, which would also alter the geometry of the *z*-dependent collective variable and the spacing between periodic membrane images.

To keep the membrane centred in the box, we retained the CHARMM-GUI restraint scheme. Position and dihedral restraints were applied with a force constant of 200 kJ mol^−1^ nm^−2^. The drug molecule was free to move along *z* to allow permeation, but to reduce lateral drift and improve reproducibility we applied a harmonic restraint in the *xy* plane. This *xy* restraint was centred on the solute's initial *x*–*y* coordinates and used a force constant of 1000 kJ mol^−1^ nm^−2^. Well-tempered metadynamics was performed using as collective variable the (unsigned) distance between the membrane centre of mass (COM) and the solute COM along the bilayer normal. Gaussian hills were deposited every 500 MD steps, with a width *σ* = 0.2 nm and an initial height of 2.4 kJ mol^−1^. A bias factor of 50 was used throughout. We used a leap-frog integrator with a timestep of 2 fs and ran each well-tempered metadynamics simulation for up to 200 ns. From the resulting free-energy profile, we estimated the permeation barrier for each candidate across each membrane proxy. Specifically, we defined the reference free energy (Δ*G* = 0) as the mean value of the free energy profile when the solute COM lay 7–8 nm from the membrane COM, *i.e.*, a region in which solute–membrane interactions are negligible. The barrier for a given membrane was then taken as the maximum Δ*G* relative to this reference. Finally, to obtain an overall barrier for each compound, we summed the barriers associated with the apical and basolateral membrane proxies.

To assess the effect of lateral heterogeneity in the lipid membrane, we repeated the metadynamics calculations for the five reference compounds using four additional restrained crossing paths in the membrane plane. In addition to the original path centred at (*x*, *y*) = (3.4, 3.4) nm, approximately the centre of the 6.8 × 6.8 nm^2^ membrane plane, we considered paths centred at (1.7, 5.1), (5.1, 5.1), (1.7, 1.7), and (5.1, 1.7) nm, corresponding respectively to the NW, NE, SW, and SE positions. These coordinates place the additional paths approximately at the quarter and three-quarter positions along each lateral box dimension. In each case, the same harmonic restraint in the *xy* plane was retained but centred on the corresponding position. These simulations therefore probe how the calculated permeation barrier depends on the local membrane environment encountered along different restrained crossing paths through the heterogeneous bilayer.

## 3 Results and analysis

### 3.1 Screening with random forests

Since our aim is to screen the full PubChem library, we generated BBB-permeability propensity predictions for all compounds that pass the preprocessing filters described in the Methods. Fig. 5 shows the distribution of the predicted probability of BBB permeation. Interestingly, whilst it is widely accepted that in the natural world most molecules cannot in fact permeate the BBB,^5–8^ we obtained a distribution roughly centred near a probability of permeation of 0.6. Aside from a modest upward shift that may reflect class imbalance in the B3DB training set, this distribution indicates that the clique-based random forest assigns intermediate probabilities to most of PubChem, rather than making confident calls at either extreme. Were the model broadly confident across this chemical space, one would expect a much larger fraction of predictions to lie near low permeability, in line with the prevailing view that BBB-permeable compounds are relatively uncommon.^5–8^ A plausible explanation is that the clique descriptor does not capture sufficient discriminative information for many PubChem structures, and that the relatively limited size of B3DB (∼7800 molecules) constrains the model's ability to generalise to a library exceeding 10^8^ compounds.

**Fig. 5:**
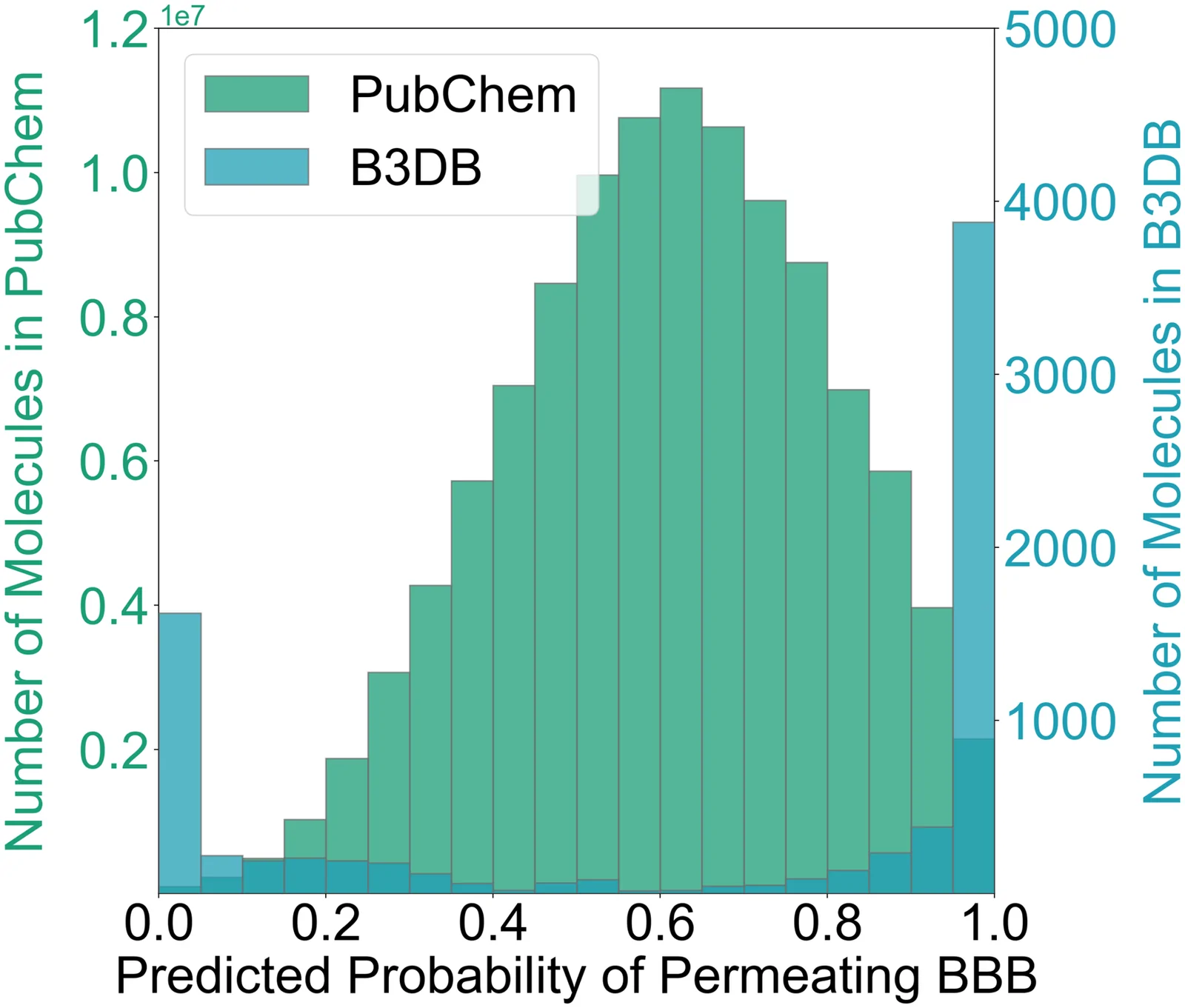
Histogram of random-forest predicted BBB permeation probability, *P*(BBBP^+^), for the screened PubChem library (left axis) compared with the B3DB training set (right axis). The B3DB predictions are strongly bimodal, consistent with the underlying BBBP^−^/BBBP^+^ labels, whereas the PubChem predictions form a mildly skewed unimodal distribution centred at intermediate probabilities. This disparity likely reflects the limited chemical-space coverage of the comparatively small B3DB training set, leading to less-confident (mid-range) probabilities when extrapolating to PubChem; see the main text for details.

To account for this, we use extremely tight classification thresholds to search for BBBP^+^ and BBBP^−^ compounds to select for molecules which we are highly confident on. This is also in part motivated by the large number of molecules present in PubChem, which gives us an abundance of potential molecules to choose from. For instance, even 0.1% of the PubChem dataset would correspond to over 100 000 molecules. As discussed in the methodology, we used a threshold of *P*(BBBP^+^) = 1.0 and *P*(BBBP^+^) ≤ 0.05 when searching for BBBP^+^ and BBBP^−^ compounds respectively. We used a slightly higher threshold for BBBP^+^ than BBBP^−^ as we found that the model is skewed towards BBBP^+^. This is likely due to the B3DB training dataset being slightly skewed towards BBBP^+^ (2850 BBBP^−^ compounds, 4950 BBBP^+^ compounds). This also allows us to achieve a high precision: in terms of metrics, recall is less valuable here, owing to the size of PubChem. Even with such strict thresholds, we still detect a high number of BBBP^−/+^ molecules. This is illustrated in Fig. 3. Despite the strict thresholds, the random forest model produces almost 100 000 BBBP^−^ and 200 000 BBBP^+^ candidates. The rates for precision and recall are shown in Table 2. We estimated these rates using repeated *k*-fold cross-validation (5 folds, 10 independent repeats). As shown in Table 2, repeated cross-validation gives precisions of 0.995 and 0.978 for the BBBP^+^ and BBBP^−^ selections, respectively.

**Table 2:** Precision and recall for identifying BBBP^+^ and BBBP^−^ molecules, estimated *via* repeated *k*-fold cross-validation on the B3DB dataset. BBBP^+^ predictions use a classification threshold of 1.0, while BBBP^−^ predictions use a threshold of 0.05

	Precision	Recall
Searching for BBBP^+^	0.995	0.304
Searching for BBBP^−^	0.978	0.478

Of the 112 194 965 PubChem molecules retained after preprocessing, 1 950 367 have BBBP^+^ scores in the range 0.95 ≤ *P* < 1.0, whereas 197 884 attain the maximum score of 1.0. We emphasise that these scores should not be interpreted as physical probabilities of BBB permeation. As shown in Table 2, the threshold of 1.0 gives a cross-validated precision of 0.995 for BBBP^+^ predictions on held-out B3DB data.

As discussed in the Methods, selecting a small subset of these candidates for MD validation is non-trivial: after filtering, the remaining compounds are broadly drug-like and carry high-confidence BBBP labels, leaving no obvious additional criterion for prioritisation. We therefore used the log BB random forest as a ranking filter to select compounds from the two candidate pools. The model is relatively weak as a quantitative regressor, with *R*^2^ = 0.494; thus, we cannot interpret the predicted log BB values or small differences between nearby ranks as physically meaningful. Instead, the purpose of said model is simply to provide a reproducible way of prioritising compounds that have already passed the BBBP classification and the subsequent filtering steps. The final 10 BBBP^+^ and 10 BBBP^−^ molecules selected for explicit validation *via* metadynamics simulations, together with their CHARMM-GUI generated parameter penalties, are reported in Table 3.

**Table 3:** PubChem compounds selected for metadynamics validation and associated parameterisation diagnostics. The first ten entries are predicted BBBP^−^ and the last ten are predicted BBBP^+^. CID is the PubChem compound identifier and formula is the (net) chemical formula used for reporting. The random-forest outputs are the classification score *P*(BBBP^+^) and the regression prediction log BB. Parameterisation was performed *via* CHARMM-GUI using CGenFF (v3.0); *μ*_pen_ and *σ*_pen_ denote the mean and standard deviation of the CHARMM-GUI/CGenFF penalty scores over generated parameters for each molecule (lower is better, with large values indicating potentially unreliable parameters). To our knowledge, these compounds do not have published BBB-permeability measurements; the table reports model-derived rankings used to define the validation set

CID	Formula	*P*(BBB^+^)	log BB	*μ*_pen_	*σ*_pen_
129065244	C_23_H_23_NO_6_	0	−0.276	25.57	26.67
161215786	C_23_H_32_NO_4_^+^	0.036	−1.168	6.81	6.50
22833297	C_29_H_40_NO_4_^+^	0.044	−1.214	29.67	24.26
23336651	C_22_H_30_NO_5_^+^	0.047	−1.086	15.93	19.31
35217964	C_22_H_30_NO_4_^+^	0.031	−1.137	12.80	13.58
44396917	C_26_H_34_NO_4_S^+^	0.008	−1.386	39.09	44.94
53822330	C_20_H_34_N_4_O_3_S	0.016	−1.221	21.45	24.52
57998458	C_30_H_38_NO_4_^+^	0.049	−1.218	8.66	9.77
6410685	C_14_H_19_N_5_O_3_S	0.023	−1.124	14.33	20.66
67197004	C_29_H_34_NO_4_^+^	0.016	−1.202	7.91	4.61
129723484	C_18_H_22_N_2_S	1	1.256	4.99	7.08
139346692	C_21_H_25_F_3_N_4_S	1	1.424	8.79	14.98
15882869	C_21_H_28_N_2_S	1	1.247	1.46	1.30
162443215	C_23_H_22_F_2_N_6_OS	1	1.188	37.60	32.60
166796104	C_17_H_17_F_3_N_2_S	1	1.320	6.91	12.78
192760	C_20_H_25_N_3_S	1	1.268	1.26	1.18
3066532	C_21_H_27_N_3_S	1	1.157	6.26	11.33
56209827	C_22_H_26_N_4_OS	1	1.198	20.59	21.59
57232718	C_25_H_29_F_3_N_2_S	1	1.273	6.60	13.38
60302500	C_20_H_24_F_2_N_2_S	1	1.263	5.28	8.08

Given the cost of the metadynamics calculations to follow, we prioritised a validation set that (i) spans the two extremes of the log BB ranking within the filtered pools and (ii) can be parametrised and simulated robustly with our CHARMM-GUI/CGenFF workflow. That combination of constraints inevitably biases the final shortlist toward chemotypes that are both common and well behaved in this parameterisation pipeline. In our case, the lowest-log BB candidates were predominantly bulky, permanently cationic structures (consistent with poor passive BBB permeation), whereas the highest-log BB candidates were enriched in neutral, lipophilic heteroaromatic scaffolds (features typically associated with higher BBB permeability). Although this reduces chemical diversity, it provides a cleaner test of the model in the regime it is actually used for because it asks whether the predicted ranking remains meaningful under modest, scaffold-level structural variations, rather than being driven by trivially different chemistry.

To assess whether the 20 compounds selected for metadynamics are structurally related to molecules represented in the B3DB training set, we used two independent molecular representations: Morgan fingerprints^53^ and MACCS keys.^54^Fig. 6a and b shows two-dimensional UMAP projections obtained from these representations. These projections provide a qualitative visualisation of the location of the selected compounds relative to B3DB and to a random sample of 100 000 PubChem molecules. More directly, Fig. 6c and d reports, for every candidate, the maximum Tanimoto similarity to any molecule in B3DB. Across the complete candidate set, the nearest-neighbour similarities span 0.198–0.610 for Morgan fingerprints (median 0.407) and 0.724–0.952 for MACCS keys (median 0.847). These results provide a qualitative assessment of the applicability domain, rather than evidence that the candidates lie within a quantitatively validated region of the ML model's feature space. Importantly, the predictive random forest uses clique descriptors, not Morgan fingerprints or MACCS keys, so nearest-neighbour similarity in these alternative representations is only a structural proxy for its domain of applicability. Moreover, the UMAP maps are two-dimensional projections and cannot establish neighbourhood relationships in the underlying high-dimensional chemical space. MACCS keys are a relatively short, dense 166-bit representation; their high Tanimoto similarities (median 0.847) are therefore not directly comparable to those from the sparser Morgan fingerprints (median 0.407, minimum 0.198), which provide a more discriminating indication of local structural differences. Several selected compounds consequently have rather distant Morgan-fingerprint neighbours in B3DB. A comparison with the already calculated single-path total permeation barriers reveals no clear monotonic relationship within either model-predicted class (Spearman rank correlations *ρ* = −0.079 for BBBP^+^ and *ρ* = −0.236 for BBBP^−^, with ten compounds per class). Given the small and deliberately selected sample, and the uncertainties of the restrained crossing paths, this lack of a detected association does not establish reliable predictions for the structurally most distant compounds. We therefore refrain from defining a strict similarity cutoff or claiming that the molecular set is entirely within the model's validated applicability domain.

**Fig. 6:**
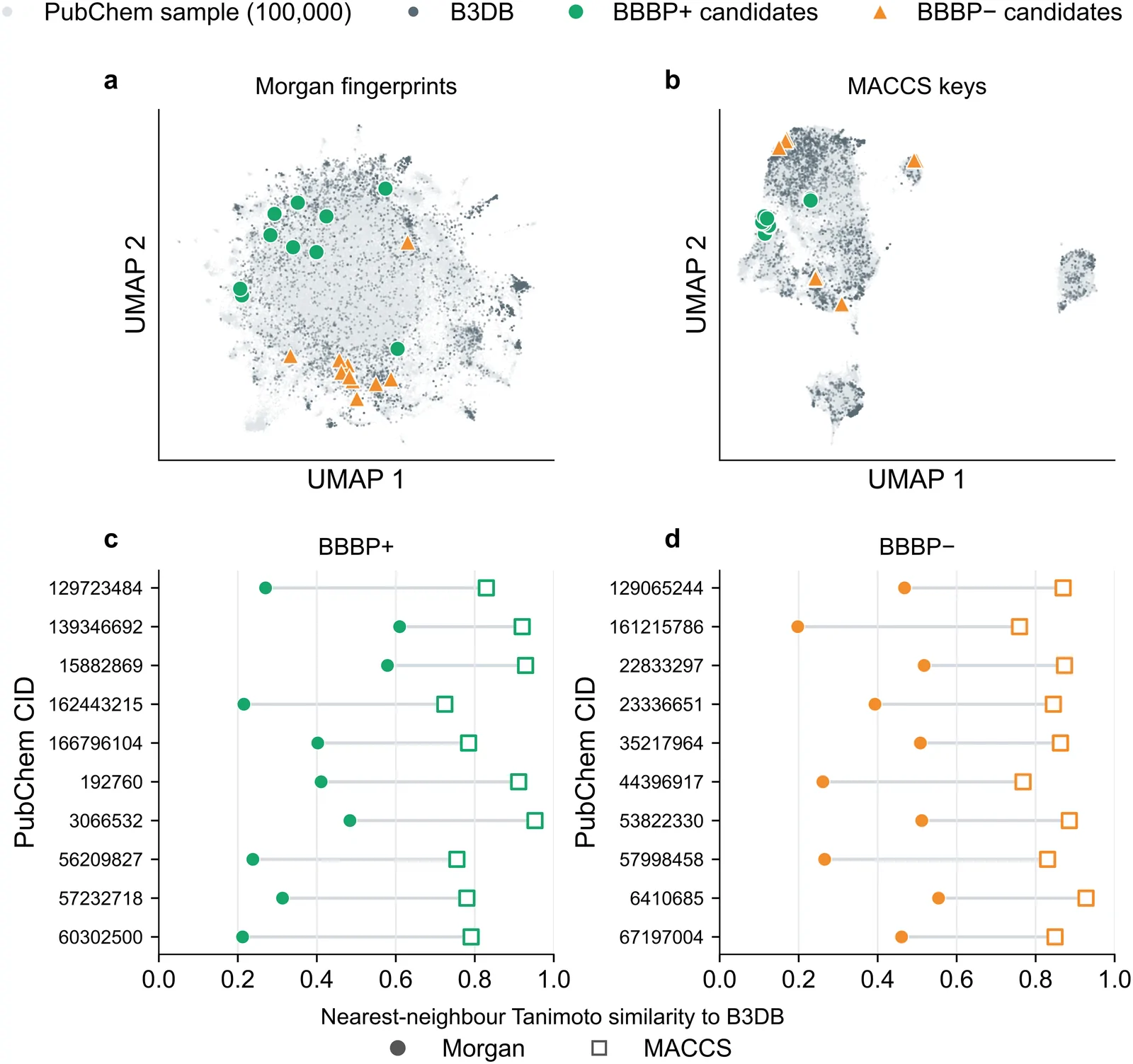
Structural relationship between the 20 candidates selected for metadynamics validation and the B3DB training set. (a and b) Two-dimensional UMAP representations constructed using Morgan fingerprints and MACCS keys, respectively. The UMAP embeddings were fitted to a random sample of 100 000 PubChem compounds, after which the B3DB molecules and the selected BBBP^+^ and BBBP^−^ candidates were projected into the same embedding. (c and d) Nearest-neighbour Tanimoto similarity of each BBBP^+^ and BBBP^−^ candidate to the most similar molecule in B3DB, evaluated independently using Morgan fingerprints and MACCS keys.

### 3.2 Well tempered metadynamics

In order to cross-validate the reliability of the model, we have decided to employ metadynamics simulations (see Methods section), which should serve to quantify directly the propensity for a given molecule to cross the BBB. Despite the relatively inexpensive computational setup we have chosen to adopt, these simulations are still characterised by a substantial computational effort, which is needed chiefly to converge the free energy profile relative to the drug molecule crossing the BBB to an acceptable accuracy. We begin by computing the free energy relative to 5 reference molecules for which we posses ground truth information on BBB permeability. This is crucial to assess whether our methodology can reliably reproduce BBBP^−^ and BBBP^+^ behavior. The free energy profiles for all 5 reference molecules across both membranes are reported in Fig. 7. As discussed in the Methods section, the collective variable of choice is the unsigned distance between the centre of mass of the drug molecule of interest and the centre of mass of the lipid bilayer proxying the BBB – along the *z* Cartesian coordinate which corresponds to the direction perpendicular to the plane in which the membrane is lying (see inset of Fig. 4).

**Fig. 7:**
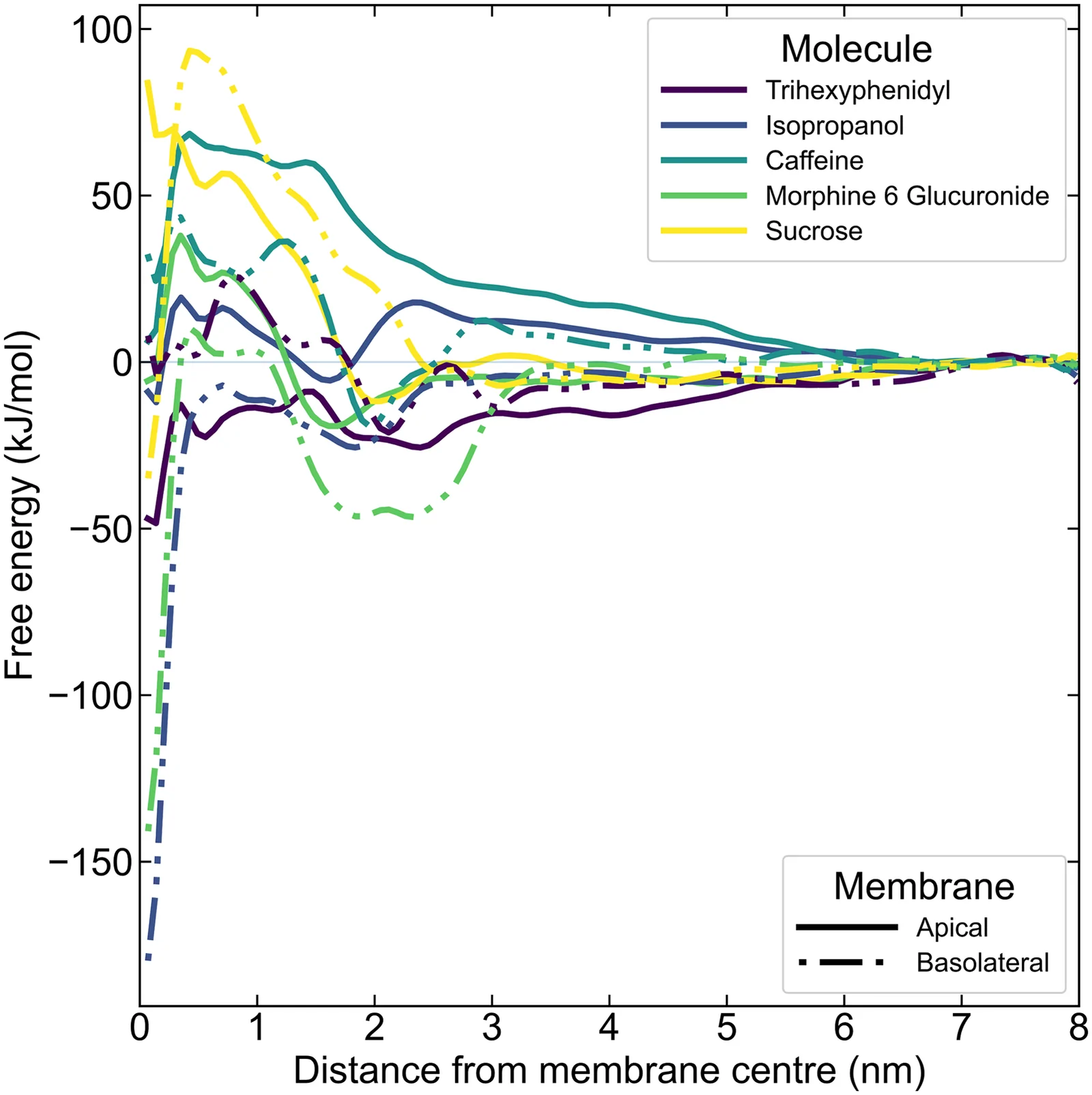
Free-energy profiles for five reference compounds as a function of their (unsigned) centre-of-mass distance from the bilayer centre along the membrane normal, obtained from well-tempered metadynamics. Solid and dot–dash lines correspond to the apical and basolateral BBB membrane proxies, respectively. The free-energy zero is set to the bulk-water reference region (*d* = 7–8 nm).

When comparing the total permeation barriers for the reference set in Fig. 7, the simulations reproduce the expected trends for most compounds. Trihexyphenidyl and isopropanol are classified as BBB-permeable in B3DB, with experimental log BB values of 1.32 and −0.10, respectively, whereas morphine-6-glucuronide and sucrose are strongly BBB-impermeable (log BB = −2.09 and −1.70).^36^ Consistent with these labels, the permeable controls exhibit relatively low total barriers, while the impermeable controls exhibit substantially higher barriers, for both bilayer compositions used to represent the apical and basolateral membranes. Caffeine is the notable exception: despite being labelled BBB-permeable in B3DB (no log BB reported, however), it exhibits a comparatively large barrier in our lipid-only models.^36^ This discrepancy likely reflects the limitations of the present setup. In particular, experimental BBB penetration metrics can be influenced by factors beyond passive transbilayer permeation, and there is evidence that caffeine transport includes a saturable carrier-mediated component in addition to diffusion.^55,56^ We therefore treat caffeine as an instructive outlier and interpret the reference set primarily as a qualitative check that the protocol captures the correct ranking for strongly permeant and strongly impermeant controls across both membrane proxies.

It is important to note that allowing the membrane area to relax does affect the absolute barrier height, and the magnitude of this effect is strongly system dependent (Fig. 8). For the ten reference compound–membrane combinations, the difference in maximum barrier between lateral-pressure-coupled and NVT simulations, Δ*B* = *B*_*xy*_ − *B*_NVT_, ranges from −67.3 to +24.9 kJ mol^−1^. Lateral relaxation reduces the maximum barrier in seven of the ten cases and increases it in three (Fig. 8). This overall tendency is consistent with the relaxation of an area constraint that can artificially elevate a barrier, but the sign and magnitude are compound and membrane dependent. The relative ordering of all five reference compounds is also not strictly preserved when the apical and basolateral barriers are summed: caffeine is particularly sensitive to pressure coupling, so its position relative to other reference compounds changes. The most strongly impermeant reference, sucrose, nevertheless retains a large summed barrier in both protocols. These results show that neither absolute barrier heights nor the detailed five-compound ranking can be assumed invariant under lateral area relaxation. We therefore retain the NVT protocol for the candidate simulations to ensure a consistent comparison, while treating the resulting absolute barriers and compound-level ordering as protocol-dependent. We separately assess the influence of local membrane heterogeneity using different restrained crossing paths, as described below.

**Fig. 8:**
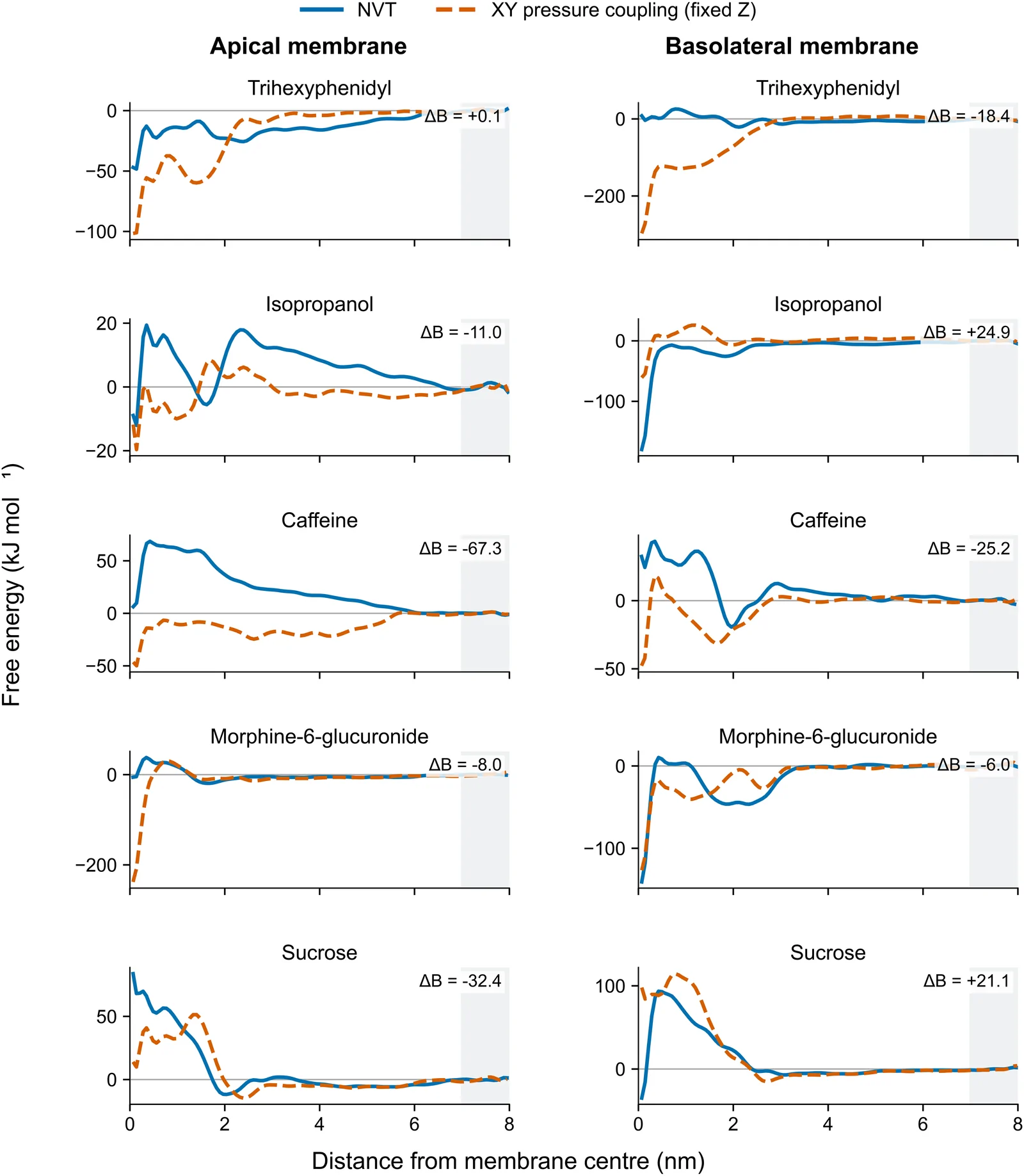
Effect of lateral pressure coupling on the metadynamics free-energy profiles for the five reference compounds across the apical (left) and basolateral (right) membrane proxies. Solid blue curves correspond to the NVT simulations used throughout this work, while dashed orange curves correspond to control simulations using C-rescale pressure coupling in the membrane plane, with an *xy* compressibility of 4.5 × 10^−5^ bar^−1^ and zero compressibility along *z*. The shaded region marks the bulk-water reference interval (7–8 nm). Δ*B* is the difference between the maximum barrier obtained with *xy* pressure coupling and the corresponding NVT value, *B*_*xy*_ − *B*_NVT_, in kJ mol^−1^.

At this stage we are well-positioned to investigate the BBBP tendency for our selection of 10 BBBP^−^ and 10 BBBP^+^. The full free energy profiles are shown in Fig. 9 and summarised in Fig. 10. We report the free energy barrier corresponding to the crossing of the drug molecule through both the apical and basolateral lipid membrane of the BBB, as well as the total energy barrier (computed as the sum of the free energy relative to the two different membranes). The free energy barrier corresponding to the crossing of the drug through each membrane is calculated as the maximum free energy required for the molecule to transit the membrane.

**Fig. 9:**
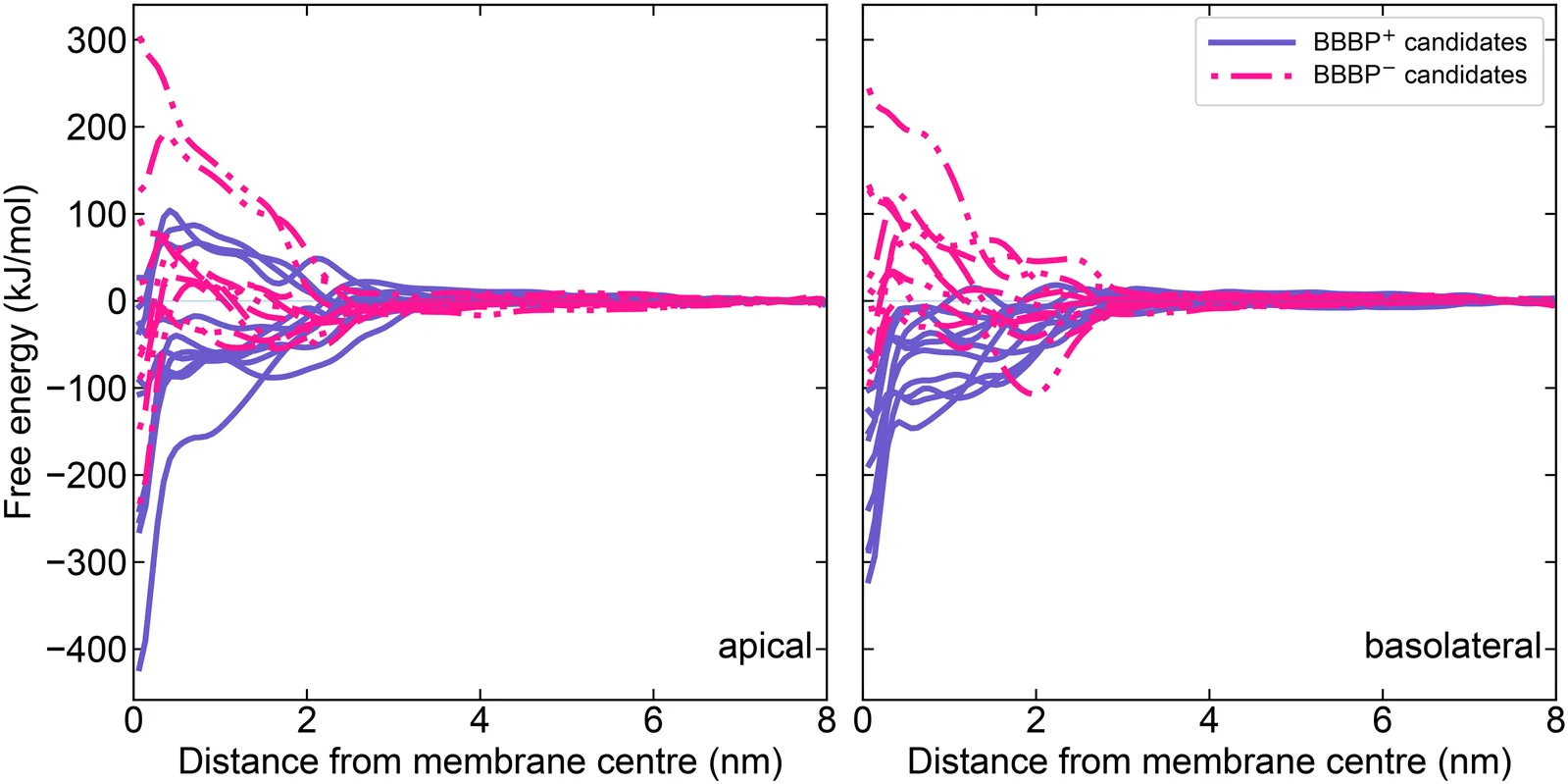
Free-energy profiles for the candidate molecules across the apical (left) and basolateral (right) BBB membrane proxies, computed *via* well-tempered metadynamics. BBBP^+^ candidates are shown as solid violet curves, and BBBP^−^ candidates as dot–dash magenta curves. The legend distinguishes the two candidate classes; individual PubChem compound CIDs and chemical formulas are reported in Table 3.

**Fig. 10:**
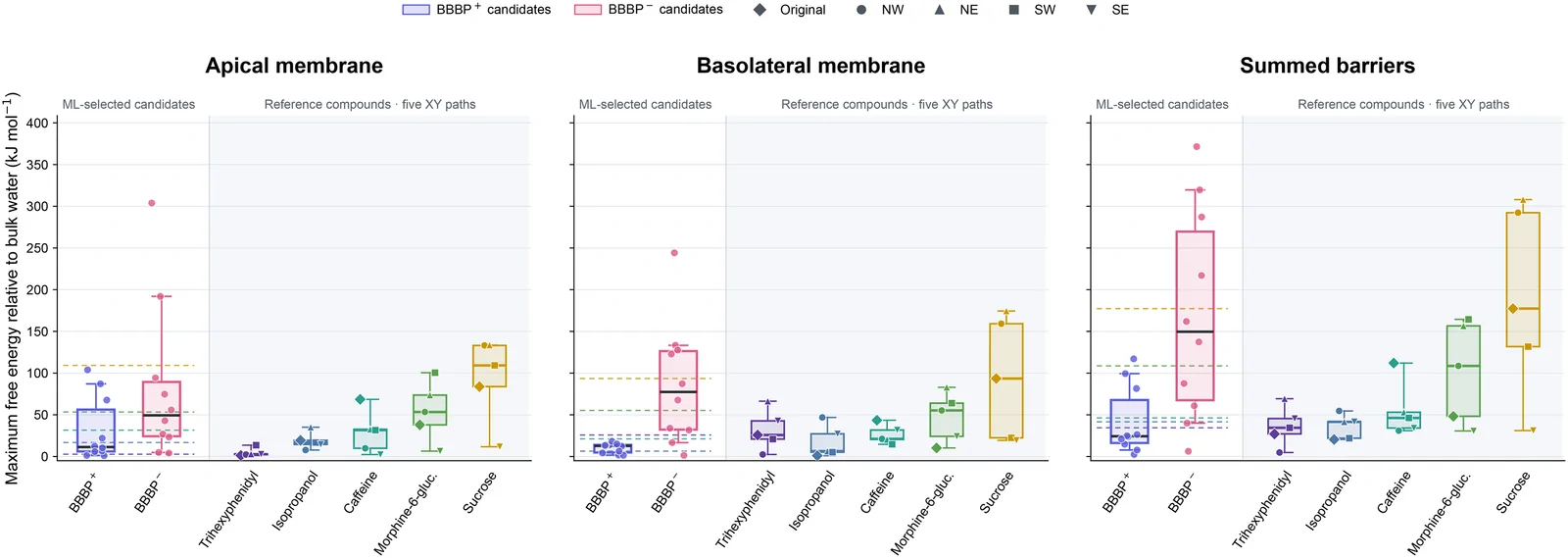
Maximum free-energy barriers obtained from well-tempered metadynamics for the candidate set and for the five reference compounds. For the reference compounds, five restrained crossing paths through the membrane plane were considered: the original central position, (*x*, *y*) = (3.4, 3.4) nm, and four additional positions at (1.7, 5.1), (5.1, 5.1), (1.7, 1.7), and (5.1, 1.7) nm (NW, NE, SW and SE, respectively). The simulation box spans approximately 6.8 × 6.8 nm^2^ in the membrane plane. The left, centre and right panels report the apical, basolateral and summed barriers, respectively. For the BBBP^+^ and BBBP^−^ candidate sets, the boxplots show variation between the 10 molecules in each class under the original restrained protocol. For each reference compound, the boxplots instead show variation between the five restrained *xy* paths. Dashed horizontal lines indicate the median barrier for each reference compound across these five paths. For the summed reference barriers, apical and basolateral values corresponding to the same *xy* position are added for display.

Fig. 9 and 10 broadly match the trends suggested by the predictions of our ML model. To assess whether the free-energy barriers differ systematically between BBBP^+^ and BBBP^−^ candidates, we apply the Mann–Whitney *U* test as a qualitative consistency check.^57^ This choice is appropriate here as the observations can reasonably be treated as independent and the free-energy barrier is a continuous (hence ordinal) variable. We formulate the test as follows. Under the null hypothesis *H*_0_, BBBP^+^ and BBBP^−^ candidates have the same distribution of permeation free-energy barriers. Under the alternative hypothesis *H*_1_, BBBP^−^ candidates have a distribution that is stochastically greater than that of BBBP^+^ candidates. We perform the test using scipy.stats.^58^ For the apical membrane alone, we cannot reject *H*_0_ at the 5% level: the test yields *U* = 69 with *p* = 0.0827. In contrast, for the basolateral membrane we reject *H*_0_ in favour of *H*_1_ at the 1% level (*U* = 89, *p* = 0.00104). The same holds for the total barrier obtained by summing both membranes: *U* = 83 with *p* = 0.00575, allowing rejection of *H*_0_ at the 1% level. Overall, these results indicate that BBBP^−^ candidates indeed tend to face larger total free-energy barriers to permeation than BBBP^+^ candidates.

Fig. 10 also shows how the calculated barriers for the five reference compounds vary when the restrained crossing path is moved to different positions in the membrane plane. The spread varies substantially between compounds, confirming that the local environment within these heterogeneous bilayers can affect the barrier associated with a particular crossing path. The spread in the summed barriers across the five restrained paths is characterised by a median spread of 8.10 kJ mol^−1^. For comparison, the difference between the median summed barriers for the BBBP^−^ and BBBP^+^ candidate sets is 125.3 kJ mol^−1^. These results illustrate that local membrane heterogeneity can introduce variations comparable to the observed class separation for some species. Importantly, however, the reference-compound spread is not a transferable per-candidate uncertainty estimate, and the Mann–Whitney result for the 20 candidates is based on a single restrained path per candidate. We therefore cannot establish from these controls that the class separation would remain statistically significant under alternative crossing paths for all candidates.

Note that all simulation results, parameters as well as analysis code are available *via* github at https://github.com/HenryJia/BBBP-MD.

A key aspect that needs to be discussed is the accuracy of our metadynamics simulations. Overall, we struck a compromise between computational efficiency and accuracy. For the candidate set, we performed 40 metadynamics simulations (20 compounds against two membrane proxies), each run for up to 200 ns, corresponding to 8 μs of production sampling, excluding equilibration and the additional reference/control calculations. That amounts to several microseconds of simulation time (excluding the equilibration protocol), which is a substantial computational effort. To test the convergence of the free energy profiles obtained *via* our metadynamics simulations, we generated a series of free energy profiles using data accumulated every 10 ns. We then calculated the mean absolute change from each free energy profile to the next. The results are shown in Fig. 11. Fig. 11 shows that the metadynamics simulations are reasonably converged for all compounds against both the apical and basolateral membrane proxies. Although each run was continued to 200 ns, the free-energy profiles stabilised after approximately 100 ns. Beyond this point, the mean change in the profile fell below 10 kJ mol^−1^. We interpret this residual drift as a practical noise floor, reflecting the intrinsic statistical fluctuations of the sampling rather than continued systematic relaxation of the free-energy estimate. We are aware that more informative methods to quantify the uncertainty related to the estimate of free energy profiles obtained by means of metadynamics simulations are available (such as reweighting-based techniques^59^). However, we note that we have used these simulations in an almost high-throughput fashion in order to cross-validate a sizeable number of prediction from our ML model – as opposed to focusing on the accurate description of the BBB crossing mechanism and the associated thermodynamic cost for a specific system in detail.

**Fig. 11:**
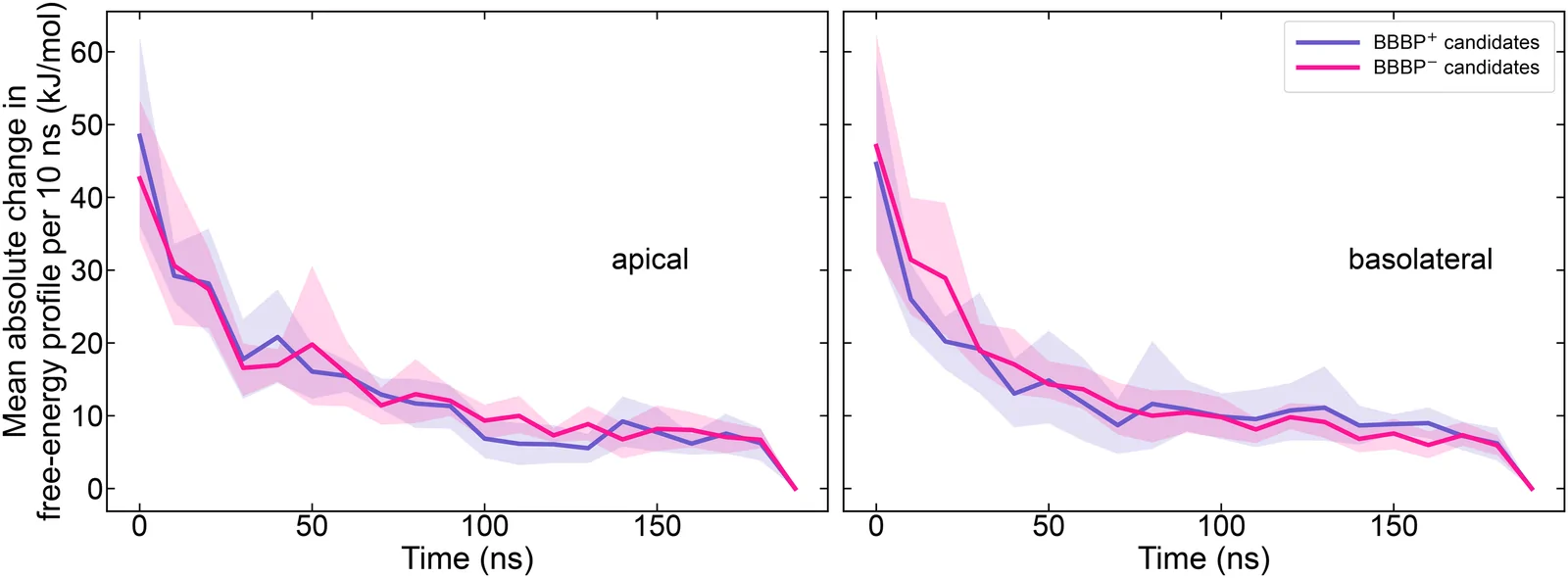
Convergence of the well-tempered metadynamics free-energy profiles for the candidate set across the apical (left) and basolateral (right) BBB membrane proxies. The mean absolute change between successive free-energy profiles accumulated over 10 ns intervals, is plotted as a function of simulation time. Solid curves report the mean over all BBBP^+^ (slate blue) and BBBP^−^ (deep pink) candidates; shaded bands indicate the 95% bootstrap confidence interval of the mean.

In addition to the permeation free-energy barriers, we also quantified how each candidate perturbs the local structural order of the lipid bilayer. Specifically, we computed the SMAC collective variable introduced in ref. 52 and 60. Although SMAC was originally developed to monitor crystal nucleation from solution,^61^ it provides a convenient and flexible measure of local molecular ordering. Here, we use it to test the hypothesis that BBBP^−^ candidates disrupt lipid ordering more strongly than BBBP^+^ candidates. For each molecule *i*, the SMAC collective variable *s*_*i*_ is defined as:
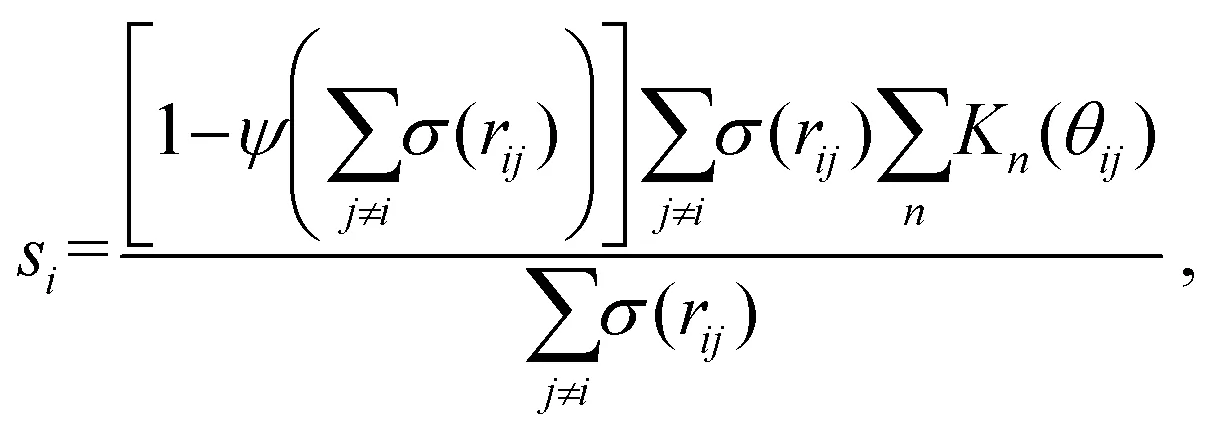
where *r*_*ij*_ is the mean distance between molecules *i* and *j*, and *σ*(*r*_*ij*_) is a switching function that restricts the sum to a neighbourhood around molecule *i*. The function *ψ* is a second switching function: the prefactor 
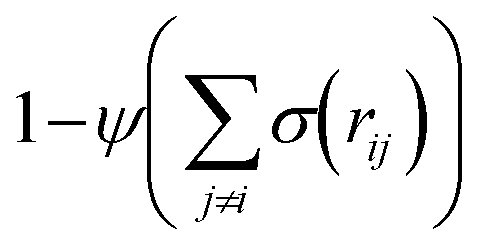
 suppresses contributions when only a small number of neighbours are present, so that *s*_*i*_ is appreciable only in locally dense environments. The functions *K*_*n*_ are kernel functions of the torsional (orientational) angle *θ*_*ij*_ between molecules *i* and *j*.

In this work, we evaluate SMAC for the lipids to probe changes in bilayer order. We use two Gaussian kernels *K*_*n*_, centred at 0 and π, each with variance (π/4)^2^. To define *θ*_*ij*_ between two lipid molecules, we select one fatty-acid tail and construct a vector from the first to the last carbon atom of that tail; *θ*_*ij*_ is then the angle between the corresponding vectors. The specific atoms used for each lipid are listed in Table 4, and a visual example for SAPS is shown in Fig. 12.

**Table 4:** Atoms selected to define the lipid-tail vectors used to compute inter-lipid angles for the SMAC collective variable. Full lipid names are given in Table 1. Atom numbering follows the CHARMM36 convention

Lipid type	SMAC starting atom	SMAC end atom
SAPS	C21	C220
SAPC	C21	C220
POPC	C21	C210
CHL1	C20	C25
OSM	C1F	C18F
SAPE	C21	C220
SOPE	C21	C218
SAPI	C21	C220
SLPC	C21	C218

**Fig. 12:**
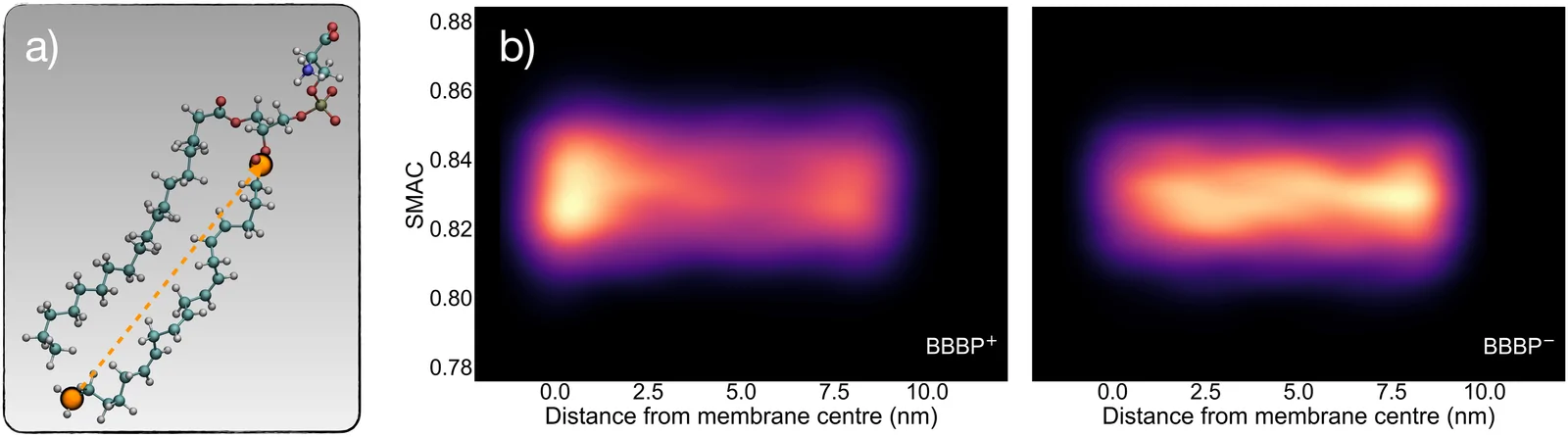
(a) Definition of the lipid-tail vector used to compute inter-lipid orientational angles in the SMAC collective variable for SAPS: the two tail atoms employed to build the vector are highlighted (orange) and connected by the dashed line. (b) Kernel-density estimate (KDE) heatmaps of the SMAC collective variable as a function of the solute distance from the membrane centre (nm), pooling configurations from both apical and basolateral simulations. The left and right panels report BBBP^+^ and BBBP^−^ candidates, respectively; colour intensity encodes the sampling density (black indicates negligible/unsampled regions).

As summarised in Fig. 12, the SMAC values span a narrow range and show no strong dependence on solute position relative to the membrane for either BBBP^+^ or BBBP^−^ candidates. Within the resolution of this analysis, we therefore do not observe a systematic shift in lipid ordering as the solute approaches or traverses the bilayer. The contour plots do differ, but this largely reflects differences in how the two classes populate the permeation coordinate: under an identical metadynamics protocol, BBBP^+^ candidates preferentially sample membrane-proximal (smaller-distance) configurations, whereas BBBP^−^ candidates remain weighted towards bulk-water (larger-distance) configurations. This separation is consistent with the free-energy barriers summarised in Fig. 10, and provides additional (albeit qualitative) support that the simulation outcomes align with the ML-based predictions. For the restrained, approximately vertical crossing paths sampled here, the SMAC analysis detects no systematic change in lipid-tail ordering associated with solute position. This observation is consistent with the prior hypothesis that chemical functionality and solute–lipid interactions can influence BBB permeation,^31^ but it is not a general demonstration that bilayer restructuring is unimportant. In particular, this setup does not allow the solute to search freely in the membrane plane for packing defects or locally favourable permeation pathways.

Because the solute remains restrained in the *xy* plane, however, the SMAC analysis probes membrane reorganisation only along the selected local crossing paths. The absence of a strong SMAC response should therefore not be taken to exclude stronger local rearrangements, or the exploitation of packing defects or local heterogeneities, for an unconstrained solute. In addition, our test set is restricted to small molecules (MW < 500); qualitatively different behaviour may emerge for larger species, such as peptides.^62^

## 4 Conclusion

In this work we have combined a transparent, fragment-based random forest ML model with atomistic molecular dynamics to explore blood–brain barrier permeability on two very different length and time scales. On the machine-learning side, we have shown that a comparatively simple cliques-based descriptor, coupled to a lightweight random forest classifier, is sufficient to screen essentially the entirety of the PubChem database for BBB-permeable (BBBP^+^) and BBB-impermeable (BBBP^−^) small molecules using only commodity CPU resources. By adopting deliberately stringent decision thresholds, we obtain high-precision classifications while still retaining several thousand candidates in each class, thus providing a practical starting point for future experimental validation and – hopefully – rational design.

Clearly, the simplicity of the clique descriptor comes with some limitations. Cliques encode which molecular fragments are present, but not their three-dimensional arrangement, stereochemistry, conformational flexibility, or the spatial distribution of charge and polarity. The fact that such a reduced representation nevertheless performs well on B3DB is quite striking, and suggests that a substantial fraction of the BBB-permeability behaviour can be captured from relatively simple information about molecular composition and functional groups. At the same time, those missing degrees of freedom might be important for the actual interaction of a molecule with a membrane.

For this reason, we have used atomistic simulations to test whether the selected compounds behave consistently with their predicted BBB-permeability class. Specifically, we have performed well-tempered metadynamics simulations for a subset of ten BBBP^+^ and ten BBBP^−^ candidates through proxy models for the blood–brain barrier, resolving permeation free-energy profiles across both apical and basolateral lipid membranes. The resulting barriers are broadly consistent with available experimental data for reference compounds and, crucially, are statistically higher for BBBP^−^ candidates than for BBBP^+^ ones when both membranes are considered together. Within the selected chemical classes and the single-path simulation protocol, this provides a physics-based consistency check on the classifier output, rather than a demonstration of external predictive validity or pathway-independent permeability.

We have further analysed the structural response of the lipid environment to monitor changes in local ordering within the membrane upon the crossing of the relevant drug molecules. Within the level of resolution offered by our simulations, we do not observe systematic differences in membrane disruption between BBBP^+^ and BBBP^−^ compounds. Given the lateral restraint imposed on the solute, however, this result applies to the restrained crossing paths sampled here and should not be interpreted as evidence that membrane reorganisation is generally unimportant during unrestricted permeation.

Several limitations of the present study are worth emphasising. First, our ML models inherit the finite size and chemical coverage of the underlying B3DB dataset and, despite the preprocessing of PubChem, will inevitably be less reliable in sparsely represented regions of chemical space. Second, our simulations probe passive permeation of neutral or ionisable small molecules through a lipid proxy of the BBB; they do not account for active transport, efflux, or interactions with proteins that are known to play a major role *in vivo*. Third, metadynamics-based free-energy profiles remain sensitive to the choice of force fields, collective variables and simulation time scales, and our validation set is necessarily limited in size.

A further limitation concerns lateral membrane heterogeneity. In the main metadynamics protocol, the solute is restrained in the *xy* plane while it permeates along the bilayer normal, so each candidate samples a particular local membrane environment. To quantify the sensitivity to this choice, we repeated the calculations for the five reference compounds from four additional lateral starting positions. As shown in Fig. 10, the resulting barriers vary with the chosen path, and the magnitude of this variation is strongly system dependent. This confirms that local composition and packing fluctuations in the heterogeneous bilayer contribute to the uncertainty of an individual barrier estimate. These controls nevertheless retain the harmonic *xy* restraint and therefore do not reproduce unrestricted lateral diffusion of the solute. A more complete treatment would require substantially broader sampling of the membrane plane, or an alternative restraint that allows the solute to search laterally for favourable permeation pathways.

Notwithstanding these caveats, the present work illustrates that a deliberately minimal ML architecture can be scaled to tens of millions of compounds and still yield classifications that are broadly consistent with membrane permeation barriers calculated for a small, deliberately selected subset under a standardised simulation protocol. We envisage several concrete extensions, including enlarging and rebalancing the training data with additional curated BBB datasets, exploring alternative descriptor sets that retain computational efficiency, and refining the enhanced-sampling protocol and collective variables for more complex chemotypes. Ultimately, we hope that the workflow outlined here will be useful as a modular component in early-stage drug-discovery pipelines, where fast, interpretable and resource-efficient BBB permeability estimates are required to prioritise candidates for more in-depth experimental and computational follow-up.

## Conflicts of interest

There are no conflicts to declare.

## Data Availability

Data for this article are available in Zenodo at [https://doi.org/10.5281/zenodo.18478319], including the blood–brain barrier permeability predictions from the PubChem screening workflow. The code and supporting simulation outputs, including molecular dynamics/metadynamics input parameters and analysis scripts, are available in GitHub at [https://github.com/HenryJia/BBBP-MD].
